# Epigenetic Regulation by Lysine Demethylase 5 (KDM5) Enzymes in Cancer

**DOI:** 10.3390/cancers3011383

**Published:** 2011-03-16

**Authors:** Lauren P. Blair, Jian Cao, Mike Ran Zou, Joyce Sayegh, Qin Yan

**Affiliations:** Department of Pathology, Yale University School of Medicine, New Haven, CT, USA; E-Mails: lauren.blair@yale.edu (L.P.B.); jian.cao@yale.edu (J.C.); ran.zou@yale.edu (M.R.Z.); joyce.sayegh@yale.edu (J.S.)

**Keywords:** KDM5, JARID1, RBP2, PLU1, SMCX, SMCY, Lid, histone demethylase, cancer stem cell, drug resistance

## Abstract

Similar to genetic alterations, epigenetic aberrations contribute significantly to tumor initiation and progression. In many cases, these changes are caused by activation or inactivation of the regulators that maintain epigenetic states. Here we review our current knowledge on the KDM5/JARID1 family of histone demethylases. This family of enzymes contains a JmjC domain and is capable of removing tri- and di- methyl marks from lysine 4 on histone H3. Among these proteins, RBP2 mediates drug resistance while JARID1B is required for melanoma maintenance. Preclinical studies suggest inhibition of these enzymes can suppress tumorigenesis and provide strong rationale for development of their inhibitors for use in cancer therapy.

## 1. Introduction

### 1.1. Epigenetics in Cancer

Stable inheritance of epigenetic states is essential for the maintenance of tissue and cell type specific functions [1]. It is now apparent that epigenetic alterations contribute significantly to tumor initiation and progression [1,2]. Epigenetic changes are reversible, raising the possibility that inhibition of specific enzymes that regulate epigenetic marks would have antitumor effects. In fact, two key families of epigenetic regulators, histone deacetylases (HDACs) and DNA methyltransferases (DNMTs), have been successfully targeted for cancer treatment [2]. For example, an HDAC inhibitor, suberoylanilide hydroxamic acid (vorinostat), has been approved by FDA for the treatment of cutaneous T-cell lymphoma [3]. Two DNMT inhibitors, 5-azacytidine (azacytidine) and 5-aza-2′-deoxycytidine (decitabine), have been approved for the treatment of myelodysplastic syndrome [4,5]. The importance of epigenetic regulation in cancer is also underscored by the findings that a number of genes encoding critical epigenetic regulators, including MLL1, MEN1, EZH2, SETD2, JARID1C, BAF180 and UTX, have been identified as mutated in various human cancers [6-13].

### 1.2. Histone Methylation and Histone Methyltransferases

In eukaryotes, DNA is packaged in the form of chromatin [14,15]. The basic building block of chromatin, the nucleosome, consists of 146 base pairs of DNA wrapped around an octamer of four core histones (H2A, H2B, H3 and H4) [16]. Histone tails are subject to a variety of posttranslational modifications that affect chromatin structure and therefore influence processes such as gene expression and DNA repair. These covalent modifications include acetylation, methylation, phosphorylation, ubiquitylation and sumoylation (reviewed in [17]).

Recent studies have highlighted the importance of histone methylation on specific lysine residues with respect to transcription. These specific residues can be mono- (me1), di- (me2), or tri-methylated (me3). Whether methylation leads to transcriptional activation or repression depends on the site and the degree of methylation. Methylation on H3K9, H3K27, or H4K20 is usually linked to gene silencing, while methylation on H3K4, H3K36, and H3K79 is generally associated with active gene expression [18]. Among these modifications, methylation of the H3K4 residue is of particular interest due to its crucial role in gene activation [19]. Specifically, H3K4me3/2 residues mark the transcriptional start sites of actively transcribed genes [20-22], while a high level of H3K4me1 is associated with enhancer sequences [23]. The importance of the H3K4 methylation marks in development and disease has been discussed in several recent reviews [24-26].

The effect of histone methylation on chromatin structure is normally mediated through the recruitment of methylation specific binding proteins. Four major protein subdomains that are capable of binding to methylated lysine have been identified: the chromodomain [27-31], Tudor domain [32-34], the WD40 repeat [35], and the PHD domain [36-39]. Many methyltransferases contain one or more of these domains and can thus serve as both “readers” and “writers” of the “histone code.”

Three families of enzymes are capable of methylating histones: the PRMT family, which methylate arginine residues, and the SET-domain containing and the non-SET-domain families which both methylate lysine residues (reviewed in [18]). These enzymes add methyl marks to lysine and arginine residues predominantly on the N-terminal tails of histones although they can also modify some core histone residues, such as H3K79.

### 1.3. Histone Demethylases

Histone lysine methylation, like many other histone modifications, is reversible. The first lysine demethylase to be discovered was lysine specific demethylase 1 (LSD1/KDM1), which demethylates H3K4 or H3K9 [40,41]. LSD1 removes these marks in a reaction that utilizes flavin as a cofactor, but its activity is limited to mono- or di- methylated substrates. Trewick and coworkers predicted the existence of a second class of Jumonji C (JmjC) domain containing histone demethylases [42]. This domain is present in many proteins that also contain other domains involved in chromatin regulation and are known, or suspected, to play roles in gene regulation. Zhang and coworkers independently identified this group of histone demethylases. They successfully purified a histone H3K36 demethylase from cells using a formaldehyde release assay to track demethylase activity. This enzyme was the first identified JmjC domain containing histone demethylase, JHDM1/KDM2A [43].

Thirty JmjC domain-containing proteins have been identified in mammals [44]. This class of proteins can be phylogenetically clustered into seven subfamilies (JHDM1, JHDM2, JHDM3/JMJD2, JARID, PHF2/PHF8, UTX/UTY, and JmjC domain only) [44]. Most JmjC domain-containing proteins have been shown to possess histone demethylase activity toward specific histone methylation marks [25,45-47]. Recently a common nomenclature for histone modifying enzymes was suggested and now lysine demethylases are abbreviated as KDMs [45,47,48]. Many studies have suggested that these demethylases could serve as oncoproteins or tumor suppressors due to their ability to sculpt the histone methylation landscape [49].

**Figure 1.**
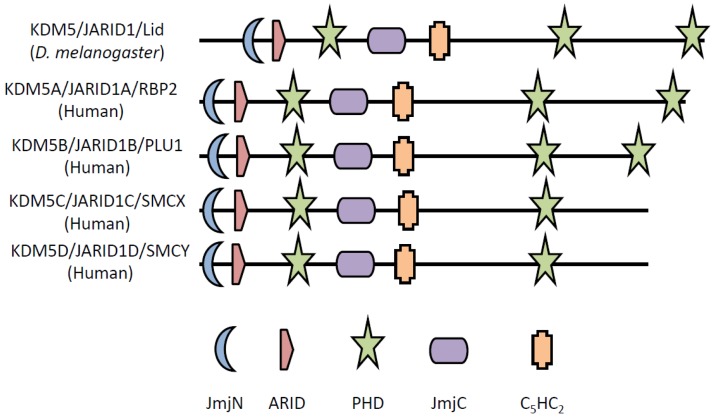
Domain structure of JARID1 proteins from *Drosophila* and humans.

This review focuses on the KDM5/JARID1 family of demethylases, which contain five conserved domains: JmjN, ARID, JmjC, PHD and a C_5_HC_2_ zinc finger (Figure 1). The KDM5 family of demethylases is represented in many different organisms including yeast and worms. The *C. elegans* KDM5 gene is called r*br-2*. Loss of r*br-2* leads to defects in vulva development [50] and decreased or increased life span [51,52]. The JmjC domain of these enzymes sets them apart from other demethylases in that it allows them to utilize a mechanism capable of removing tri- and di-methyl marks from H3K4. Their PHD domains can bind certain methylated residues allowing them to recruit other proteins, such as HDACs, to the site of methylated histones. For these reasons they can serve as both “readers” and “erasers” of the “histone code” [53].

### 1.4. Histone Methylation in Cancer

It is increasingly clear that alterations in histone methylation play important roles in cancer [18,49,54]. The presence or absence of methyl marks on certain histone residues is very important to gene expression and has many implications in cancer progression. Aberrant methylation is thought to contribute to excessive proliferation of cells and therefore to tumorigenesis. Esteller and coworkers showed that a common hallmark of human cancer is loss of the trimethylation mark on lysine 20 and the acetylation mark on lysine 16 of histone H4 [55]. Additionally, the H3K4me0 state in combination with H3K27 acetylation has been associated with poor prognosis of breast cancer [56].

Misregulation of histone lysine methylation can have negative effects on development and has been shown to contribute to many cancers [57-60]. Additionally, the role of histone methyltransferases and histone demethylases in development has been well noted and may contribute to the establishment of cancer stem cells [24,61,62]. Genome-wide analyses of chromatin states of embryonic stem cells and progenitor cells suggest that genes important for developmental control are marked by “bivalent marks”, which include both the active H3K4me3 and the repressive H3K27me3 marks [21,22,63]. Although the existence of these “bivalent marks” is currently debated, they provide a logical model of a gene being poised for transcriptional activation or repression during development. Dysregulation of these chromatin marks could change the properties of the stem cells and progenitor cells and impair their differentiation potential, which could result in cancer initiation and progression. Thus, the enzymes that regulate these modifications likely play important roles in cancer.

There are several examples in current literature that are consistent with the idea that enzymes capable of maintaining histone methylation are important in cancer. For example, MLL1, the catalytic subunit of an H3K4 methyltransferase complex, is frequently translocated in leukemia [58,59] and another H3K4 methyltransferase subunit MEN1, has been shown to be frequently mutated in endocrine tumors [9,60,64]. Additionally, EZH2, the catalytic subunit of an H3K27 methyltransferase polycomb repressive complex 2 (PRC2) [57], is overexpressed in advanced prostate cancer [65]. EZH2 activates oncogenes Ras and NF-κB and triggers metastasis by epigenetic silencing of the tumor suppressor DAB2IP/AIP1 [66-68]. Finally, genomic alterations (amplification or deletion) and/or point mutations of several histone methyltransferses and demethylases are increasingly being identified in cancers through the use of high-density SNP arrays and deep sequencing technologies [8,10-13,69,70]. For example, inactivating mutations of UTX, an H3K27 histone demethylase, were identified in multiple cancer types, including multiple myeloma, esophageal squamous cell carcinoma, renal cell carcinoma, myeloid leukemia, breast and colorectal cancers, and glioblastoma [8,12]. It has recently been shown that methyltransferases and demethylases act in concert to regulate both activating and repressive marks on histones in a dynamic process [71]. This review focuses on a class of enzymes capable of removing H3K4me3/2 marks and their potential as targets for cancer therapies.

## 2. *Drosophila* KDM5/JARID1/Lid

**Figure 2.**
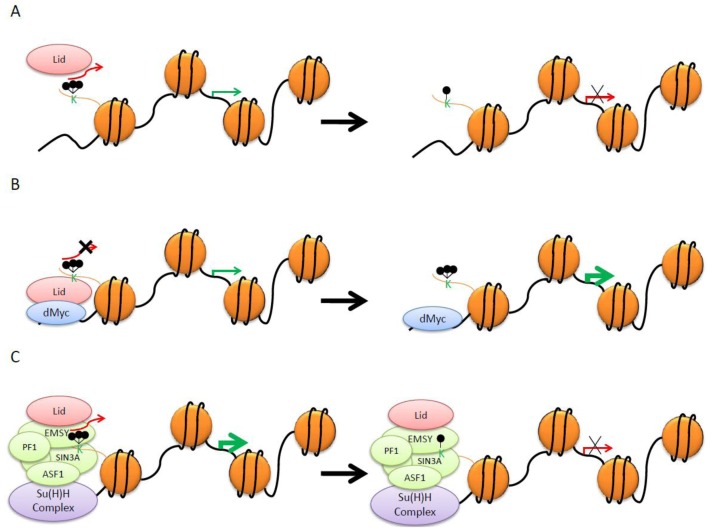
**(A)** The demethylase capabilities of Lid can contribute to gene repression by removing H3K4me3 marks, which are associated with active genes [73-75]. **(B)** Binding of dMyc to Lid inactivates its demethylase activity but retains H3K4me3 binding ability, allowing it to recruit dMyc to actively transcribed genes [76,77]. **(C)** Lid represses Notch target genes by associating with the LAF complex and demethylating H3K4me3 [78,79].

The only known JARID1 protein in *Drosophila melanogaster* is Lid, named for the phenotype seen in mutant larvae (Little Imaginal Discs) (Figure 2A) [72]. Lid has recently been classified as an H3K4me3/2 demethylase and it shares all the domains of the human JARID1 family (Figure 1) [73-75]. Because Lid is the only JARID1 family protein in *Drosophila*, it has provided an excellent model for studying the role of this class of enzymes in gene expression and tumorigenesis. Studies on Lid have shown that its knockdown in fly cells results in an increase in global H3K4me3 levels [73,75]. Like RBP2 and JARID1B, Lid has three PHD fingers which conventionally bind methylated lysines (Figure 1). Recently Li *et al.* probed the binding capabilities of Lid PHD domains in detail and discovered that the PHD1 domain of Lid binds all methylated forms of H3K9 and unmethylated H3K4 [76]. They also discovered that the PHD3 domain binds di and tri-methylated H3K4 and suggested that Lid uses this binding to recruit dMyc to regions of active transcription (Figure 2B) [76].

Myc is a transcription factor that is known to regulate the expression of roughly 15% of genes [77]. Activation of the Myc gene by amplification or translocation causes cancer [77]. The Eisenman lab performed a genetic screen to identify mutations that compensated for overexpression of dMyc, the *Drosophila* homolog of human Myc. In this screen, they discovered two independent mutations of *Lid* that suppress the rough eye phenotype caused by dMyc overexpression, suggesting that *Lid* and *dMyc* interact genetically [74]. In contrast to the notion that an H3K4me3 demethylase represses gene expression, Lid was shown to antagonize gene silencing by allowing the expression of the homeotic gene Ubx [80]. Lid is also required to maintain H3 acetylation, which is associated with open chromatin, through a mechanism that negates the deacetylase activity of Rpd3 [80,81]. Secombe *et al.* found that Lid physically interacts with dMyc, and this interaction abolishes the ability of Lid to demethylate H3K4me3 marks but allows Lid to maintain its ability to bind these marks using its PHD3 domain (Figure 2B) [74]. They also discovered that Lid facilitates dMyc binding to E-boxes, regions on DNA commonly bound by transcription factors. These findings suggest that in the presence of dMyc, Lid likely acts as a “reader” of H3K4me3 to recruit dMyc to activate gene expression, but not as an “eraser” as its demethylation activity is inhibited by dMyc (Figure 2B).

**Figure 3.**
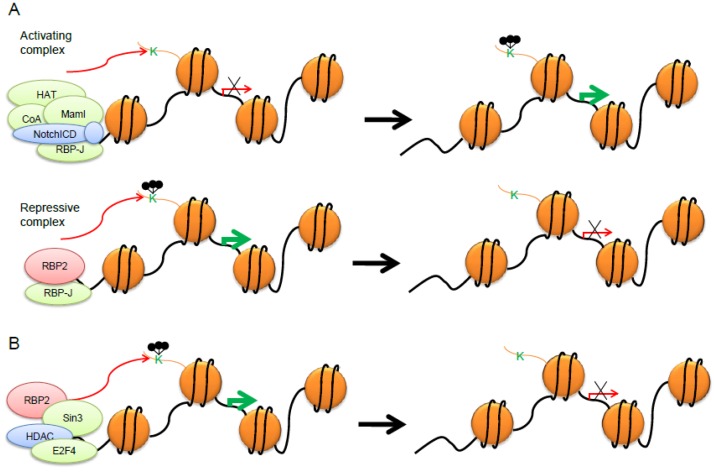
**(A)** NotchICD activates genes by recruiting RBP-J and a histone acetyltransferase (HAT) complex that adds activating marks to histones, therefore allowing transcription on Notch target genes. In the absence of NotchICD, RBP2 can bind RBP-J and demethylate existing activating marks, leading to repression of Notch target genes [83]. **(B)** RBP2 has been shown to interact with the Sin3/HDAC complexes which are crucial targets for anti-cancer therapies [88-90].

Recently, several labs have linked Lid with Notch signaling. Notch is a transmembrane protein that, upon binding its ligands, is cleaved. Upon cleavage, Notch intracellular domain (ICD) acts as a transcription factor activating many different genes (Figure 3A). When overexpressed Notch serves as an oncogene; however, it can act as a tumor suppressor in other contexts [82]. Many cancers have aberrant Notch signaling cascades and Notch has been linked to some specific cancers such as T-cell acute lymphoblastic leukemia (T-ALL) [79]. The Notch system is highly conserved between humans and flies [82].

NAP1 and ASF1 are *Drosophila* histone chaperones and are components of the complexes RLAF-N and LAF-A respectively. These complexes regulate H3K4me3 and H3 acetylation levels at Notch target genes and are required for silencing of Notch genes that are crucial to development in all metazoan systems. Moshkin *et al.* discovered that ASF1 co-purifies with Lid and that Lid can interact physically with both ASF1 and NAP1 [78]. They suggest that ASF1 aids in the silencing of Notch genes by providing a link between the LAF complex and the Su(H)/H co-repressor complex. In this complex, Lid aids in gene repression by demethylating H3K4me3 at Notch genes (Figure 2C). They propose a similar mechanism for the involvement of Lid in the RLAF-N complex. A report by Liefke *et al.* has probed the interaction between Lid and Notch even further by establishing that Lid physically interacts with Su(H), and with recombinant RBP-J, the mammalian homolog of Su(H) [83]. They show that mutation or reduced expression of Lid enhances tumorigenesis and growth related to Notch signaling in flies [83]. Due to the high degree of conservation between human and fly signaling pathways, Lid provides an excellent model system for elucidation of the role of KDM5 enzymes in cancer.

## 3. KDM5A/JARID1A/RBP2

RBP2, also called KDM5A or JARID1A, was initially isolated as a binding partner of retinoblastoma protein (pRB) [84]. The gene coding for pRB, *Rb1*, is a well documented tumor suppressor gene that is frequently inactivated, directly or indirectly, in a wide variety of cancers [85]. pRB not only inhibits cell cycle progression by blocking S-phase entry, but also promotes differentiation and senescence. The ability of pRB to promote differentiation and senescence tightly correlates with its ability to bind to RBP2 and, in many models, this phenomenon can be recapitulated by inhibiting RBP2 with siRNA [86]. Consistent with these observations, RBP2 can repress certain genes that are activated by pRB [86]. Interestingly, genome-wide location analyses indicated that RBP2 binding sites are also enriched for E2F binding sites, suggesting that the functions of pRB in cell cycle control and differentiation are inter-connected [87].

In 2007, RBP2 was discovered to function as an H3K4me3/2 histone demethylase [50,88]. Our report outlining the catalytic activity of RBP2 also noted that deletion of RBP2 causes increased H3K4me3 at the SDF1 promoter [88]. Restoration of the methylation state requires that RBP2 have an intact JmjC catalytic domain [88]. It has also been determined that, mechanistically, RBP2 promotes cell growth and inhibits senescence and differentiation. In a mixed genetic background, *RBP2* knockout mice are viable and grossly normal [88]. Using this knockout mouse model, we showed that loss of the RBP2 gene causes decreased apoptosis and increased G1 entry of the cell cycle in hematopoietic stem cell and myeloid progenitor cell compartments [88]. Additionally, knockdown of RBP2 in SAOS-2 osteosarcoma cells leads to upregulation of cell cycle regulators p21, p27 and p130 [86]. Consistent with these results, complete loss of RBP2 leads to increased expression of p27 [88].

Other studies have shown that RBP2 is overexpressed in gastric cancer [91]. RBP2 triggers cellular senescence of gastric and cervical cancer cells through binding to the promoters of cyclin-dependent kinase inhibitors p16, p21, and p27 and removing tri-methylated H3K4 at these sites [91]. Complete abrogation of RBP2 from mouse cells inhibits cell growth, induces senescence and differentiation, and causes loss of the “stemness” property of embryonic stem cells *in vitro*. Moreover, loss of RBP2 dramatically inhibits tumorigenesis in a mouse cancer model [92]. These findings suggest that RBP2 is an ideal target for cancer therapy in multiple cancer types.

In addition to pRB, RBP2 interacts with many proteins involved in oncogenesis, including p107, TBP [93], LMO2 [94], nuclear receptors [95], Myc [74], Sin3/HDACs [88-90], Mad1[96], and RBP-J [83]. The fact that RBP2 interacts with nuclear receptors and enhances expression of their target genes [86,95] suggests that RBP2 could recruit additional factors important for nuclear receptor-mediated transcription, which plays important roles in breast and prostate cancer progression. Of special interest is the interaction of RBP2 with Myc, which is often activated in cancers as mentioned above [74]. A recent report, however, indicated that RBP2 is recruited by Mad1 to a Myc target gene hTERT. By removing the activating histone marks at the hTERT promoter, RBP2 represses hTERT expression when associated with Mad1 [96]. The Lid/Notch relationship mentioned above is conserved in humans with relation to RBP2. Liefke *et al.* report that RBP2 is an essential component of the Notch/RBP-J repressor complex and that it is necessary for removal of H3K4me3 at RBP-J sites [83]. This group also showed that RBP2 binds RBP-J in place of NotchICD when Notch cleavage is inhibited (Figure 3A) [83]. This leads to a decrease in H3K4me3 and a subsequent suppression of Notch target genes (Figure 3A). Taken together, RBP2 can suppress Notch signaling through histone demethylation and could be crucial to the suppression of Notch-induced tumorigenesis.

In acute myeloid leukemia patients RBP2 has been shown to form a fusion protein with a nuclear pore complex protein, NUP98 [97]. This fusion retains the third PHD finger of RBP2, which binds H3K4me3 marks on histones. Overexpression of this fusion protein alone is sufficient to arrest hematopoietic differentiation and induce acute myeloid leukemia in murine models [98]. RBP2-NUP98 can bind to and prevent the removal of H3K4me3 at promoters of many lineage-specific transcription factors and thus increase gene expression. Mechanistically, it serves to prevent the demethylase activity of KDM5s and the methyltransferase activity of the PRC2 complex and allows for methylation patterns distinct to leukemic stem cells [98].

A recent study suggested that increased expression of RBP2 promoted a stem cell like phenotype and enhanced resistance to anti-cancer agents by changing chromatin structure [99]. The Settleman lab generated erlotinib resistant versions of PC9 lung cancer cells, which are normally erlotinib sensitive, by exposing these cells to media containing increasing concentrations of erlotinib [99]. Interestingly, they showed that the resistance could be reversed after withdrawing the drug, suggesting that erlotinib resistance arises from changes of epigenetic states of these cells [99]. In the erlotinib resistant population of PC9 cells, RBP2 expression was increased, leading to decreased global levels of H3K4me3 and H3K4me2. Moreover, they showed that RBP2 is required to maintain the drug tolerant state of PC9 cells [99]. RBP2 has previously been shown to be associated with HDAC activity (Figure 3B) [88-90], therefore the Settleman lab set out to determine the effect of HDAC inhibitors on drug resistant PC9 cell lines. They found that upon treatment with HDAC inhibitors, drug resistant cells were rendered drug sensitive [99]. This provides strong rationale for development of small molecule inhibitors of RBP2 to overcome common drug resistance.

## 4. KDM5B/PLU1/JARID1B

Another KDM5 family protein found in humans, JARID1B (also referred to as KDM5B and PLU1), was discovered in experiments targeting genes regulated by a tyrosine kinase, HER2 [100]. Cells overexpressing HER2 were treated with the antibody 4D5 (Herceptin) which inhibited phosphorylation of HER2. JARID1B was identified as one gene remarkably downregulated by Herceptin treatment. Northern blotting was then used to show that JARID1B is consistently expressed in breast cancer cell lines, but is expressed at restricted levels in normal adult tissues with the exception of the testis [100]. Barrett *et al.* verified the expression of JARID1B in breast cancer cell lines and in primary tissues and discovered that 90% of invasive ductal carcinomas express JARID1B [101]. Other studies involving JARID1B and breast cancer have shown that it is a transcriptional repressor that physically interacts with developmental transcription factors BF-1 and PAX9 [102]. Barrett *et al.* performed an extensive analysis of the interaction between JARID1B and HDAC4 [103]. They found that two of the PHD domains of JARID1B interact with the N-terminal tail of HDAC4 and that JARID1B co-localizes with HDAC4 to matrix-associated deacetylase bodies [103].

The exact enzymatic function of JARID1B was unknown until 2007 when several groups classified it as a histone demethylase [50,104,105]. Yamane *et al.* showed that JARID1B represses tumor suppressor genes such as BRCA1, CAV1 and 14-3-3σ and that knockdown of JARID1B increases H3K4me3 at these target genes [104]. Interestingly, they were able to demonstrate that downregulation of mouse JARID1B suppresses mammary tumor growth in a syngeneic mouse cancer model. This suggests that JARID1B could be a prime target for breast cancer therapies [104]. Consistent with the limited gene expression pattern of JARID1B, *JARID1B* knockout mice are viable [106], and will be a valuable tool to study its role in mammary development and breast cancer.

JARID1B has also been shown to be a potential oncogene in other cancers such as prostate, lung, bladder and melanoma [107,108]. Using data from the Oncomine database [109,110] as well as frozen tissues, Xiang *et al.* showed that JARID1B is up-regulated in prostate cancer samples while showing limited expression in the benign prostate. These up-regulations have been noted at both the mRNA and the protein levels [108]. One explanation for this phenomenon might be the interaction of JARID1B with the androgen receptor (AR) which enhances AR-dependent transcriptional activity [108].

Hayami *et al.* recently showed that at both the protein and mRNA levels, JARID1B is up-regulated in tumor tissues of bladder cancer and lung cancer (both SCLC and NSCLC) [107]. They also noted that knockdown of JARID1B induces growth suppression in cell lines derived from these cancers [107]. Further analysis of JARID1B knockdown cell lines led them to conclude that transcription factors E2F1 and E2F2 are downregulated when JARID1B expression is downregulated [107]. These findings further support those found by the Zhang lab suggesting that JARID1B downregulation leads to suppression of tumor formation, which makes it an ideal drug target for many different cancers [104].

JARID1B has also been suggested as a potential target for anti-cancer vaccines [111]. Liggins *et al.* discovered that JARID1B is one of the non-X cancer-testis (CT) antigens. These types of proteins are of interest due to their limited expression in normal tissues but overexpression in many kinds of cancer. Additionally, they can induce a cytotoxic T-lymphocyte response which makes them prime candidates for cancer vaccine development. Among a panel of CT antigens screened in B- and T-cell malignancies, JARID1B is highly expressed in both cases at the mRNA level [111]. Indeed, in breast cancer patients, there are T cells that react with HLA-A*201* peptides of JARID1B [112].

JARID1B expression has previously been shown to be expressed at higher levels in melanocytic nevi than in advanced and metastatic melanomas [113,114], but its role in melanoma was unclear until recently. Roesch *et al.* found that increased JARID1B expression marks a slow cycling population of melanoma cells [113]. This cell population is associated with prolonged growth and self-renewal potential in serial transplantation experiments. This study also noted a link between JARID1B and Notch signaling by showing that JARID1B represses the Notch ligand Jagged 1. This repression leads to less Notch cleavage and a subsequent decrease in expression of Notch target genes [113]. These results suggest that, in melanoma, JARID1B may not be required for cancer establishment but that it most likely contributes to tumor progression and metastasis. Furthermore, they found that expression of JARID1B is dynamically regulated, which suggests that stemness of melanoma cells could be dynamic [113].

## 5. KDM5C/JARID1C/SMCX

Another KDM5 family protein, JARID1C (also known as KDM5C and SMCX), has mostly been studied in context of mental retardation but has been linked to some forms of cancer [105,115-117]. Human papillomavirus (HPV) is thought to be the leading cause of cervical cancer and the second most common cause of cancer death in women worldwide [118]. The long control region (LCR) of HPV contains oncogenes E6 and E7 which can be repressed when bound by E2. In an effort to determine genes involved in the tumor suppressor capabilities of E2, the Howley lab performed an siRNA screen. This screen identified JARID1C as a mediator of the HPV E2 tumor suppressor protein [119]. They then determined that JARID1C is physically recruited by the E2 protein to repress the transcription of the oncoproteins E6 and E7 through the HPV LCR [119].

Clear cell renal cell carcinomas (ccRCC) are traditionally classified as having mutations in the VHL gene and activation of the HIF pathway [120,121]. In an effort to discover other mutations in ccRCC as part of the Cancer Genome Project, the Sanger Institute underwent an extensive gene expression analysis of a ccRCC tumor panel. This screen led to the discovery that 3% of ccRCC tumors contain truncation mutations in JARID1C [8]. They also noted that JARID1C is associated with hypoxic tumors rather than non-hypoxic tumors and that most tumors with JARID1C mutations also contained VHL mutations [8]. JARID1C was shown to be a HIF target gene in RCC cells, but it suppresses tumor growth, suggesting that inactivation of both VHL and JARID1C is required for tumor formation in this subtype of ccRCC [122].

## 6. KDM5D/JARID1D/SMCY

The least well documented member of the KDM5 family, JARID1D (also referred to as KDM5D and SMCY), is coded for by the SMCY gene, which is located on the Y chromosome. JARID1D, like other KDM5 family members, is capable of demethylating di- and tri-methyl H3K4 [75,105,123]. While there is no direct link between JARID1D and cancer, a deletion analysis of Y chromosome specific genes in human prostate cancer revealed that 52% of cases showed deletion of the JARID1D gene [124]. This study associated loss of specific Y chromosome genes with prostate cancer, suggesting a role for JARID1D in the pathogenesis of the disease.

## 7. Summary

In recent years much has been discovered about histone demethylases and their roles in cancer. In this review, we have chosen to focus on the role of KDM5 family members in cancer, specifically, due to their ability to regulate an activating mark on H3K4. One of the primary hallmarks of cancer is its limitless replicative ability, much of which is acquired through overexpression of oncogenes and repression of tumor suppressor genes. KDM5 family members are capable of removing the H3K4me3 activating mark from histones which makes them potential players in the downregulation of tumor suppressors, but could also suggest that their activity could repress oncogenes. These activities indicate that KDM5 family members could be prime targets for many different therapies in a context dependent manner. Indeed these enzymes have been suggested to have oncogenic properties in some tissues and to have tumor suppressor functions in others (Table 1). Therapies targeting this family of enzymes are currently under development by many groups.

The term “cancer stem cell” (CSC) has been defined as cancer cells within a given tumor that are capable of re-forming the tumor [126]. These cells are thought to remain after other tumorigenic cells die off during most cancer therapies. These remaining cancer stem cells can re-develop into a more aggressive tumor that is resistant to therapies. Treating cancer stem cells could provide a potent method for treating these more aggressive cancers and preventing recurrence. It is generally thought that cancer may arise from a progenitor cell that becomes a cancer stem cell or from a stem cell that becomes cancerous. The connection between epigenetic regulation and development suggests that epigenetic changes may be the first step in cancer progression. In fact, it was proposed that cancer stem cells arise through epigenetic changes [1]. A clear understanding of how histone demethylases act in stem cell development may give us hints of how to eliminate cancer stem cells. Specific to the KDM5 family proteins, drug resistant cells overexpressing RBP2 seem to have some features found in cancer stem cells [99] and JARID1B marks a subpopulation of human melanoma cells that can sustain tumor growth and self renewal, suggesting a new definition of melanoma stem cells [113].

One class of histone modifying enzymes that has been successfully targeted for cancer therapy is HDACs [3]. Many histone demethylases, including RBP2 [88-90], JARID1B [103] and JARID1C [116], have been shown to interact with the HDAC complexes. Additionally, recent studies suggest that demethylases are linked to HDACs through the Sp1 protein [127]. This link was discovered when the Chen lab noted that cells treated with HDAC inhibitors appeared to also be downregulated for KDM5 demethylase activity. These studies suggest that success of HDAC inhibitors could be correlated to their interactions with the KDM5 enzymes. HDACs generally act on many different histone residues, while the catalytic activity of the KDM enzymes is limited to specific histone residues. This suggests that KDM inhibitors are likely to have more specific biological effects and therefore be more specific anti-cancer epi-drugs than HDAC inhibitors.

In this review we have covered many different angles for development of anti-cancer therapies using the KDM5 family of demethylases. This family of enzymes can be crucial in the expression and repression of oncogenes and tumor suppressor genes and can themselves serve as both. They also provide insight into the cancer stem cell model and how we might develop drugs targeting these cells. Some of the limitations in drug discovery thus far have been the lack of appropriate readouts for demethylase activity and the similarity of mechanism between all proteins of this class. Recently, more accurate demethylase assays have been developed and high throughput screening techniques have been optimized for identifying specific inhibitors of these enzymes [128,129]. These inhibitors/epigenetic drugs will have the potential to reprogram cancer cells or cancer stem cells with the same gusto as the HDAC inhibitors and could have a revolutionary effect on the future of cancer treatment.

## Figures and Tables

**Table 1.** KDM5 family demethylases in cancer.

**Demethylase**	**Cancer type**	**Notes**	**Potential role**

**KDM5A/RBP2/JARID1A**	gastric	overexpressed [91]	oncogene
	leukemia	NUP98 fusion [97]	oncogene
	cervical	overexpressed [125]	oncogene
	lung	drug tolerance [99]	oncogene

**KDM5B/PLU1/JARID1B**	breast	overexpressed [100]	oncogene
	prostate	overexpressed [108]	oncogene
	bladder	overexpressed [107]	oncogene
	lung	overexpressed [107]	oncogene
	melanoma	tumor progression [113]	oncogene

**KDM5C/SMCX/JARID1C**	cervical	mediator of human papillomavirus protein E2 [119]	tumor suppressor
	kidney	inactivating mutations [8]	tumor suppressor

**KDM5D/SMCY/JARID1D**	prostate	deleted [124]	tumor suppressor
